# Characterizing Conformational Change of a Thermoresponsive Polymeric Nanoparticle with Raman Spectroscopy

**DOI:** 10.3390/s23125713

**Published:** 2023-06-19

**Authors:** Luis Trabucco, Savannah Heath, Jonathan Shaw, Sean McFadden, Xiaodu Wang, Jing Yong Ye

**Affiliations:** 1 Department of Biomedical Engineering and Chemical Engineering, The University of Texas at San Antonio, San Antonio, TX 78249, USA; luis.trabucco@utsa.edu (L.T.); savannah.heath@utsa.edu (S.H.); jonathan.shaw@my.utsa.edu (J.S.); sean.mcfadden@my.utsa.edu (S.M.); xiaodu.wang@utsa.edu (X.W.); 2 Department of Mechanical Engineering, The University of Texas at San Antonio, San Antonio, TX 78249, USA

**Keywords:** Raman spectroscopy, thermoresponsive polymer, conformational change, nanoparticles

## Abstract

Molecular conformational changes in the collapsing and reswelling processes occurring during the phase transition at the lower critical solution temperature (LCST) of the polymer are not well understood. In this study, we characterized the conformational change of Poly(oligo(Ethylene Glycol) Methyl Ether Methacrylate)-144 (POEGMA-144) synthesized on silica nanoparticles using Raman spectroscopy and zeta potential measurements. Changes in distinct Raman peaks associated with the oligo(Ethylene Glycol) (OEG) side chains (1023, 1320, and 1499 cm^−1^) with respect to the methyl methacrylate (MMA) backbone (1608 cm^−1^) were observed and investigated under increasing and decreasing temperature profiles (34 °C to 50 °C) to evaluate the polymer collapse and reswelling around its LCST (42 °C). In contrast to the zeta potential measurements that monitor the change in surface charges as a whole during the phase transition, Raman spectroscopy provided more detailed information on vibrational modes of individual molecular moieties of the polymer in responding to the conformational change.

## 1. Introduction

**Figure 1:**
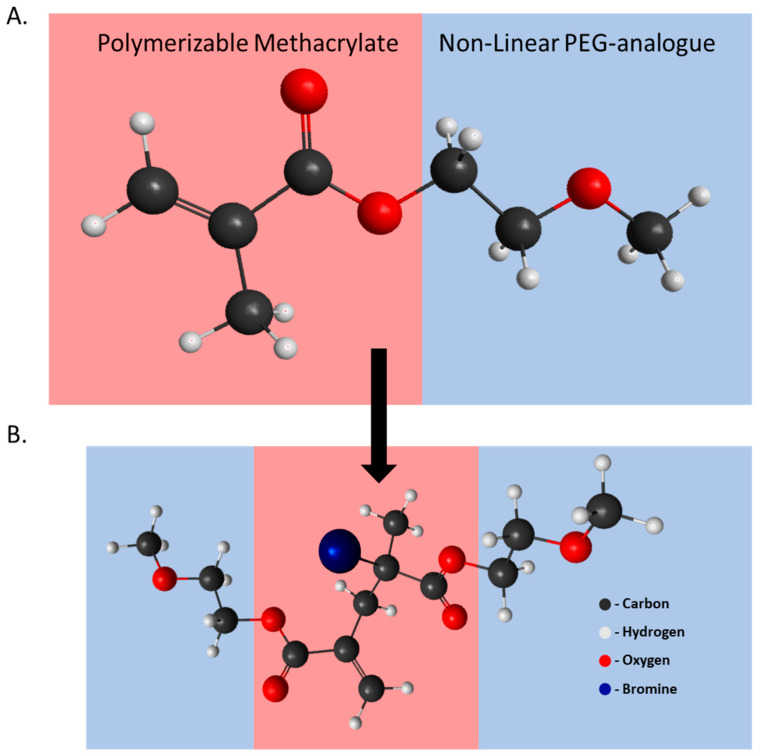
Polymerization scheme for POEGMA-144. (**A**) OEGMA monomer that is composed of a polymerizable MMA backbone (pink) and a non-linear PEG-analogue side chain (blue): Carbon (black), Hydrogen (grey), and Oxygen (red). (**B**) POEGMA polymer capped with Bromine (blue) following ATRP polymerization.

Recent advances in polymer nanoscience have led to the development of thermoresponsive polymers with the potential for a multitude of biomedical applications. These types of polymers fall within a special category of “smart” nanotechnology that can be tuned by several means of external stimulation, such as temperature, pH, ionic concentration, and irradiation. Smart polymers have been extensively utilized in numerous biomedical applications, such as drug delivery, hydrogels, and scaffolds supporting cell growth [1,2]. Within the class of thermoresponsive–smart polymers, self-healing polymers, such as Poly(oligo(Ethylene Glycol) Methyl Ether Methacrylate), i.e., POEGMA brushes, have shown significant promise in nano-biosensing and modulated drug delivery [3,4]. POEGMA brushes are composed of a polymerizable methyl methacrylate (MMA) backbone, with non-linear, oligo- poly(Ethylene Glycol) (PEG)-analogue (OEG) side chains (Figure 1). When POEGMA is in aqueous conditions at room temperature, the OEG side chains are hydrophilic and in an expanded state; however, once the local temperature rises beyond a side chain’s molecular-weight-dependent critical temperature, termed as the lower critical solution temperature (LCST), the OEG group undergoes a conformational change, collapsing in towards the MMA backbone [5,6]. This conformational change results in a phase transition that creates a new hydrophobic condition of the polymer and can be further exploited in nanoparticle–polymer conjugates [7,8]. Unlike other thermoresponsive polymers, such as Poly(N-isopropylacrylamide) (PNIPAM), that undergo a similar phase transition but do not recover to their original state after cooling, POEGMA has the unique ability to repetitively collapse and reswell when heated and cooled across its LCST [9,10,11].

Although the bulk conformational change of POEGMA brushes have been previously characterized by our group via a photonic crystal biosensor, as well as by other groups with mechanisms such as atomic force microscopy (AFM) and quartz crystal microbalance dissipation (QCM-D) [3,5,6,7,12], the corresponding molecular change associated with the conformational change is still poorly understood. Given that POEGMA’s conformation and hydrophilicity is dependent on temperature, a comprehensive chemical characterization of this conformational change in the hydrated condition is imperative for further applications in nanosensing and polymeric–nano conjugates for drug delivery. Studies have been reported to explore the molecular characteristics of the polymers through nuclear magnetic resonance (NMR) and Fourier-transform infrared (FT-IR) spectroscopy [13,14]. In addition, as Raman spectroscopy requires minimal sample preparation and is highly sensitive to both physical and chemical changes [15], it is expected to serve as an ideal tool for comprehensive studies of molecular behavior under the conformational change.

Raman spectroscopy has previously been used to characterize several thermoresponsive polymers at their transition temperatures, including PNIPAM [11,16]; however, no such characterizations exist for the conformational change/phase transition of POEGMA, to the authors’ knowledge. Previous studies showed Raman spectroscopy being utilized to confirm the incorporation of silver nanoparticles into anti-fouling POEGMA brushes for analyzing enhanced antibacterial activities rather than for characterization of conformational changes of the polymer itself [17,18]. In the study reported here, we synthesized POEGMA-144 brushes on silica nanoparticles (SNPs) and conducted Raman spectroscopy in a temperature range across its LCST to characterize the conformational collapse and reswelling of POEGMA-144. Multiple observed Raman peaks showed different temperature dependence. This study revealed the changes in the vibrational modes of the molecular moieties of POEGNA-144 during its phase transition.

## 2. Materials and Methods

In this study, we synthesized POEGMA-144 grafted silica nanoparticles (SNPs) (POEGMA@SNPs) and analyzed their thermoresponse behavior in the temperature range from 34 °C to 50 °C. Raman spectroscopy was utilized to characterize the polymer collapse about the theoretical LCST at 42 °C of POEGMA-144. Zeta potentials of the synthesized POEGMA@SNPs were measured at temperatures below and above the LCST of POEGMA-144. While both the Raman and zeta potential measurements showed clear evidence of the polymer collapse above its LCST, they provided complementary information. The zeta potential measurement reported the change in the particle surface charge property as a whole, while the Raman spectroscopy provided more detailed information of the effects of the phase transition on different moieties of the side chains and backbone of the polymer. 

### 2.1. Materials

Silica nanoparticles were purchased from Skynanoparticles and used as received, without additional purification. Trimethoxy-silylpropyl 2-Bromomethyl Propanoate (BPME) ATRP Initiator, Copper(I) Chloride, and 2-2′ Bipyridine (Bpy) were purchased from TCI America and used as received. Oligo(Ethylene Glycol) Methyl Ether Methacrylate) OEGMA-144 monomer was purchased from Sigma and purified via inhibitor removal column (SIGMA 306312). 

### 2.2. BPME Initiator Assembly onto SNPs

**Scheme 1:**
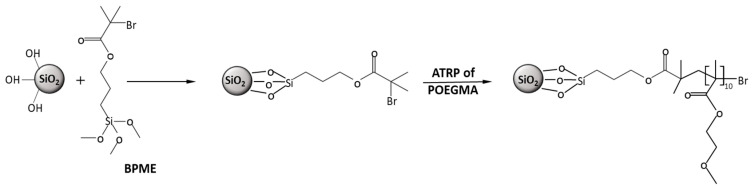
Assembly scheme for conjugations of POEGMA@SNPs. The initiator BPME is attached to SNPs via silanization, followed by ATRP of POEGMA-144.

BPME was used as an Atom Transfer Radical Polymerization (ATRP) initiator for POEGMA brush grafting from the SNPs. BPME was chosen for its α- and ω-terminals of trimethoxy silane and 2-Bromo-2-methylpropanoate, respectively. The α-terminal is responsible for polymer attachment to a specific substrate, silica in this case, while the ω-terminal is responsible for donating a free radical during polymerization to extend the polymer chain of POEGMA. The BPME immobilization procedure of silanization (Scheme 1) was adopted from a previously reported protocol [6]. Briefly, a 2 mg/mL aqueous solution of SNP was exposed to Ultra Violet Light-C (UVC light) (700 mA) for 1 min to activate surface hydroxyl groups on the SNPs. Next, BPME was introduced dropwise to the SNP solution to a final concentration of 5.66 mM and allowed to react for 16 h under gentle stirring. After this time, the Trimethoxy-silane α-terminal of the BPME initiator was bound to the surface of the SNPs to form BPME@SNP. Excess initiator was removed from the sample by a double wash in methanol via centrifugation (8500× *g* × 20 min).

### 2.3. Surface-Initiated Atom Transfer Radical Polymerization of POEGMA-144

POEGMA-144 polymer brushes were synthesized from the BPME initiator via Surface-Initiated Atom Transfer Radical Polymerization (SI-ATRP) in this polymerization reaction based on a previously described protocol [12]. Briefly, 1.99 mg of copper (I) chloride (Cu(I)Cl) was combined with 6.36 mg of Bpy and purged under a constant stream of N_2_ for 1 h. Simultaneously, POEGMA-144 monomer was added to a final concentration of 1.55 M, with 2 mL of Methanol (MeOH) and BPME@SNPs and purged with N_2_ for 1 h. Once purged, the two reactive containers were combined with a stir bar to form a dark brown yet translucent monomer solution that reacted for 30 min while spinning at 70 rpm and continuous nitrogen purging. The solution was then sealed and allowed to further react for 24 h.

Upon completion of the polymerization, the solution turned a bright blue color indicating oxidation of the Cu(I)Cl species and completion of the reaction. The synthesized POEGMA@SNPs were then purified of unused reactants by centrifugation 4× *g* in MeOH and 2× *g* in deionized water. The POEGMA@SNPs (Scheme 1) were finally resuspended in DI water and stored for characterization.

### 2.4. Instruments for Characterization of POEGMA@SNPs

A JOEL JEM-ARM 200F transmission electron microscope (TEM) was used to image POEGMA@SNPs. Zeta potentials were measured with a Zetasizer Nano ZS system (Malvern Instruments Ltd., Malvern, UK). Raman spectra were obtained using a Horiba LabRAM HR Evolution-confocal Raman spectroscopy system. Temperature control was achieved by use of an ITC-106RH Inkbird PID Thermostat controller. 

### 2.5. Preparation for Raman Spectroscopy and Thermal Heating Apparatus

Samples were prepared for Raman spectroscopy via centrifugation and removal of the supernatant, leaving a highly concentrated pellet that was resuspended in 50 µL DI water. The samples were then loaded into a heating apparatus and affixed to the translation stage of the Raman system. For the temperature-dependent study, 30 µL silicon wells with Raman-inert, conductive aluminum bottom surface was connected to a proportional– integral–derivative (PID) controller that remotely controlled the local temperature of the sample. A thermal probe was attached to the top of the heating block to monitor the samples temperature in real time and create a feedback loop to control the heating rate. 

### 2.6. Raman Characterization of POEGMA-144 Conformational Change at LCST

A continuous laser beam was focused to a spot size of ~7.5 µm in diameter to the bottom of the 30 µL well through a 10X objective (NA = 0.25, 154 mW) with an excitation wavelength of 532 nm. Ten Raman spectra with a 3 s acquisition time were taken between 200–4000 cm^−1^. Raman scans were taken at increasing and decreasing temperatures between 34–50 °C at 2 °C steps. The experiment was conducted in triplicate. 

### 2.7. Raman Spectral Processing

The 200–2000 cm^−1^ range was extracted from entire spectra, and background fluorescence was removed using a 5th-order polynomial baseline. Then, spectra were processed with a 2nd-order Savitzky–Golay (S-G) algorithm with window size of 20 to smooth the spectra by fitting a polynomial curve to the spectral data within a set window size. 

### 2.8. Selection of Raman Peaks Characteristic to POEGMA-144 Conformational Change at LCST

OEGMA macromers can be broken down into two easily definable subsections (Figure 1). First, the backbone consists of an MMA monomer that continuously propagates during polymerization via free radicals. The second section of OEGMA consists of a tunable, non-linear PEG analogue that varies in molecular weight (and length) on the basis of applications. This is the subsection that is expected to undergo a conformational change and is primarily responsible for the phase transition at the LCST. Transition temperatures increase with side chain length/molecular weight. Characteristic Raman peaks of polymethyl methacrylate (PMMA) and PEG have been used to identify subsections of POEGMA; however, much of the polymer is composed of CH_2_ and CH_3_ sections, making clear identification of peaks a non-trivial task [11,17,19,20,21]. In this study, relative changes in distinct peaks associated with OEG side chains (1023, 1320, and 1499 cm^−1^) with respect to MMA backbone (1608 cm^−1^) were evaluated. Observed spectrum assignments were based on previous studies of POEGMA [21] and its derivatives (PEG [22] and PMMA [23,24]).

Polymer collapse and reswelling were characterized via linear-corrected integrated area (IA) ratios of the OEG side chains/MMA backbone (1035 cm^−1^/1608 cm^−1^, 1323 cm^−1^/1608 cm^−1^, etc.) at every temperature tested. The linear corrected IA were calculated and utilized to minimize the effects due to variance in sample fluorescence. As the 1608 cm^−1^ peak corresponded to the sp^2^ hybridization of carbon and was a property unique to the backbone of POEGMA [25], it was used as the normalization factor. Temperature profiles for each IA ratio were generated for comparisons.

### 2.9. Statistical Analysis

For zeta potential measurements, one-tailed paired *t*-tests were conducted to validate polymerization and to assess statistical significance of polymer collapse once heated over its LCST. The Raman spectroscopy measurements were conducted in three rounds, allowing us to take an average at each temperature and calculate the standard errors. A percent reduction was calculated for the 1023 cm^−1^, 1320 cm^−1^, and 1499 cm^−1^ peaks normalized to the 1608 cm^−1^ peak from the backbone below and above the LCST temperature. The values were obtained and analyzed for both the heating and cooling phases.

## 3. Results and Discussion

### 3.1. Characterization of POEGMA@SNPs with Zeta Potential Measurements

**Figure 2:**
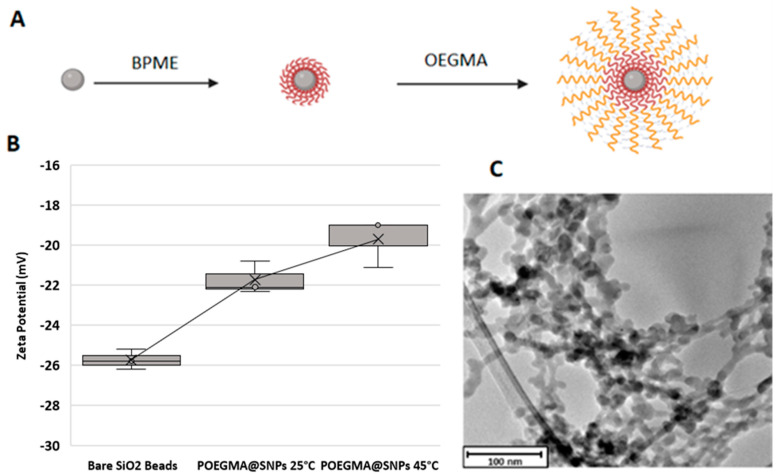
Characterization of POEGMA@SNPs. (**A**) The synthesis process for grafting POEGMA from SNPs. (**B**) Different zeta potentials of uncoated SNPs and POEGMA@SNPs, signifying a successful coating. Changes in zeta potentials of POEGMA@SNPs at the temperatures below and above its LCST were also observed. (**C**) TEM image of POEGMA@SNP network.

The SI-ATRP process of POEGMA *grafting from* the SNPs allows for a highly controllable polymerization rate and uniform coating on the grafting substrate [26,27,28,29]. The silica atom transfer radical polymerization (SI-ATRP) process is illustrated in Figure 2A. The result from the zeta potential measurements confirmed the successful grafting of POEGMA on SNPs (Figure 2B), while Figure 2C shows the TEM image of POEGMA@SNP network. The zeta potential of POEGMA@SNPs was measured as −21.5 mV, which was changed from a zeta potential of −25.5 mV for bare silica nanoparticles in an aqueous solution. This shift in zeta potential confirmed the presence of POEGMA on the surface of the SNPs. 

The zeta potential of POEGMA@SNPs was measured at 25 °C and 45 °C as representative temperatures below and above the LCST, respectively. After heating the sample to 45 °C beyond the LCST (42 °C for POEGMA-144), a shift in the zeta potential was observed from −21.5 mV to −19.5 mV (*p* = 0.0341 based on a one-tail statistical *t*-test). The change in the zeta potential may have been attributed to the inherent change in steric hindrance of the polymer as the phase transitioned from hydrophilic to hydrophobic for the side chains of POEGMA. The change in the zeta potential was relatively small, which may have been limited by the short side chain length (OEGMA-144 monomer) used in this study. It was to be expected that with longer side chains (e.g., OEGMA-300, or OEGMA-2000), a larger difference in zeta potential would be seen during the phase transition, as there would be a larger difference in steric hinderance between the side chains. Nevertheless, the change in the zeta potential observed here below and above the LCST indicated the POEGMA collapse due to the phase transition. 

### 3.2. Determination of Characteristic Raman Peaks of POEGMA@SNPs

**Figure 3:**
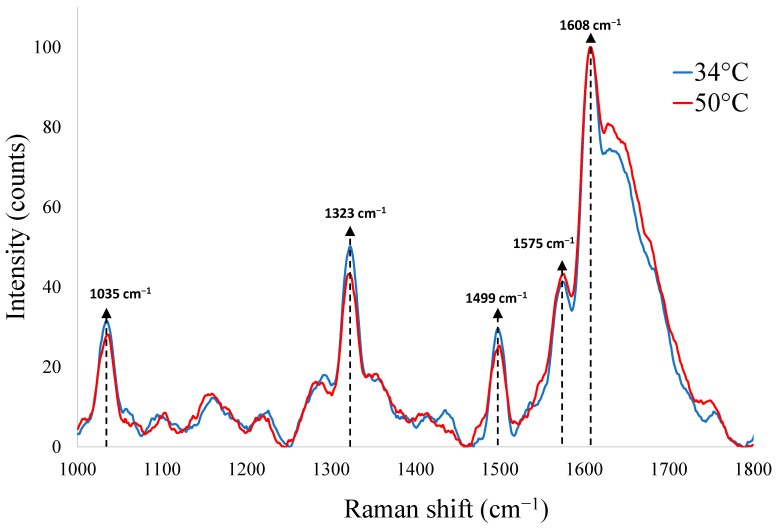
Averaged representative Raman spectra of an aqueous solution of POEGMA@SNPs at 34 °C (blue) and 50 °C (red), respectively. Changes with the peaks at 1035 cm^−1^, 1323 cm^−1^, and 1499 cm^−1^ normalized to the 1608 cm^−1^ backbone peak were apparent above and below the LCST.

The primary range of interest was determined to be 1000–1800 cm^−1^. Initial Raman scans of POEGMA@SNPs displayed five characteristic peaks that could be associated with POEGMA, as the Raman signal from the pure SNPs as a control did not show significant peaks compared with the Raman signal strength from POEGMA@SNPs within this spectral range. The characteristic peaks 1035 cm^−1^, 1323 cm^−1^, 1499 cm^−1^, 1575 cm^−1^, and 1608 cm^−1^ are shown in Figure 3. As the broad water signal in the 1500–1700 cm^−1^ range overwhelmed the 1575 cm^−1^ CH peak signal, it was not further investigated; instead, we focused on the other four peaks. 

The integrated area (IA) under the curve of the characteristic peaks of 1035 cm^−1^, 1323 cm^−1^, and 1499 cm^−1^ were normalized to that of the 1608 cm^−1^ peak. The reason for choosing the 1608 cm^−1^ peak for the normalization was because this peak could be assigned to the sp^2^ hybridization of carbon, which is a property unique to the backbone of POEGMA that is not expected to vary with the temperature [25].

### 3.3. Raman Detection of Conformational Change

**Figure 4:**
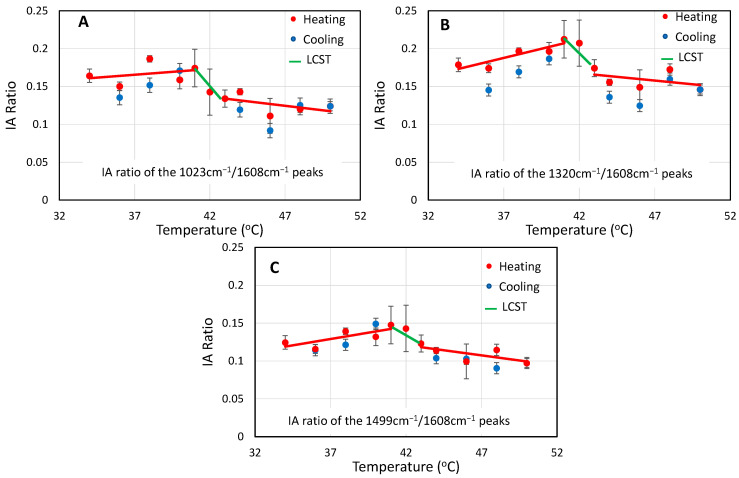
Ratios of integrated areas (IA) of different Raman peaks from the side chains over the 1608 cm^−1^ peak from the backbone of the POEGMA. (**A**) IA ratio of 1023 cm^−1^/1608 cm^−1^, (**B**) IA ratio of 1320 cm^−1^/1608 cm^−1^, and (**C**) IA ratio of 1499 cm^−1^/1608 cm^−1^ peaks measured at different temperatures. The Raman spectroscopy measurements were conducted in both heating (red) and cooling (blue) processes. The drop of the IA ratio around the LCST is marked in green.

Raman spectroscopy detects vibrational modes of molecular bonds; thus, the change in Raman signals can be associated with specific types of molecular interactions. POEGMA@SNPs displayed several characteristic peaks that were analyzed to determine the Raman signal as a function of temperature to investigate the conformational change of the polymer due to the phase transition. With the peaks at 1035 cm^−1^, 1323 cm^−1^, and 1499 cm^−1^ normalized to the 1608 cm^−1^ backbone peak, we found visible changes in the IA ratio as a function of the temperature (Figure 4). POEGMA@SNPs were measured over a range of increasing and decreasing temperatures to observe the collapse and reswelling of the polymer. For the heating process, all the three IA ratios for the 1035 cm^−1^, 1323 cm^−1^, and 1499 cm^−1^ peaks increased slowly with increasing temperature before reaching the LCST (42 °C). A clear drop of the IA ratio was observed around the LCST, as indicated with a green line in Figure 4. After the temperature passed the LCST, the IA ratio began to decrease gradually with an increase in the temperature, in sharp contrast to the behavior in the temperature range below the LCST. The change in the temperature dependence of the Raman signals indicated the phase transition of POEGMA. Since POEGMA brushes went through a conformational change at the LCST, the brushes curled towards the carbon backbone [5,6,16]. The side chain changed into hydrophobic from hydrophilic when the temperature was above the LCST. This phase transition resulted in the changing hydrogen bonding between the side chain and the surrounding water molecules, which may have been responsible for the observed changes in the Raman signals from different vibrational modes of individual molecular moieties of the side chain when the temperature passes the LCST. In addition, Figure 4 also shows the IA ratios in the cooling process. It can be seen that the Raman signals recovered when the sample was cooled below its LCST. This experimental finding indicated that the polymer collapse during the heating phase above its LCST was reversible in the cooling phase below its LCST, signaling a reswelling of the polymer. 

Previous studies of the phase transition of various types of POEGMA and copolymerized OEGMAs have been reported in detail with different techniques, such as QCM-D, FT-IR, differential scanning calorimetry (DSC), dynamic light scattering (DLS), and ultraviolet–visible spectroscopy (UV-vis). Piechocki and Kozanecki characterized the FT-IR vibrational modes of several lengths of POEGMA side chains in hydrogels as a function of the hydration state [30]. Additionally, Ramírez-Jiménez et al. investigated the effects of copolymerizing OEGMAs with various side chain lengths on cloud point and glass transition temperatures via UV-vis spectroscopy and DSC, respectively [31]. Lutz et al. also explored the thermal behavior of P(MEO2MA-co-OEGMA) hydrogels via DLS and turbidimetry [32]. The results presented in this study further the knowledge associated with POEGMA on the individual molecular moieties associated with the phase transition at the LCST. Our results are also in consistence with a recent report that investigated the conformational change of POEGMA using a QCM-D [6]. POEGMA brushes grafted from the surface of a QCM-D sensor showed the reversible phase transition nature of the coating. The QCM-D technique detected the conformational change on the basis of monitoring the dissipation factor that is primarily related to the viscoelasticity of the sample [33]. In contrast, Raman spectroscopy allows the study of the dynamic thermoresponse of individual molecular moieties of the polymer, thus reporting a different aspect of the phase transition of POEGMA for the temperature crossing its LCST. 

## 4. Conclusions

This paper reports the study of thermoresponsive POEGMA grafting from SNPs via Raman spectroscopy. The temperature dependence of multiple Raman peaks corresponding to different molecular moieties of the polymer was obtained and analyzed, which clearly indicated a phase transition of POEGMA at its LCST. In addition, zeta potential measurements were conducted to detect the change in surface charges of the synthesized POEGMA@SNPs at temperatures below and above the LCST of POEGMA-144, which also showed a phase transition in agreement with the results from the Raman spectroscopy. This study demonstrated the possibility of utilizing Raman spectroscopy for characterizing the thermoresponsive properties of POEGMA in detail for designing future applications that require molecular specificities. This study also gave rise to the need to specifically identify the Raman peak assignments of the subregions of both the polymerizable MMA backbone and the non-linear PEG analogue side chains found within POEGMA-144 and its derivatives.

## Data Availability

Not applicable.
